# The pattern of human papillomavirus infection and genotypes among Nigerian women from 1999 to 2019: a systematic review

**DOI:** 10.1080/07853890.2021.1938201

**Published:** 2021-06-14

**Authors:** Anthony Uchenna Emeribe, Idris Nasir Abdullahi, Maisie Henrietta Etukudo, Idongesit Kokoabasi Isong, Anthony Ogbonna Emeribe, Justin Onyebuchi Nwofe, Chikodi Modesta Umeozuru, Buhari Isa Shuaib, Odunayo Rahmat Oyetola Ajagbe, Amos Dangana, Bibiana Nonye Egenti, Peter Elisha Ghamba

**Affiliations:** a Department of Medical Laboratory Science, Faculty of Allied Medical Sciences, University of Calabar, Calabar, Nigeria; b Department of Medical Laboratory Science, Faculty of Allied Health Sciences, Ahmadu Bello University, Zaria, Nigeria; c Department of Medical Laboratory Science, Ebonyi State University, Abakaliki, Nigeria; d Nigeria Field Epidemiology and Laboratory Training Programme, African Field Epidemiology Network, Abuja, Nigeria; e Antiretroviral Therapy Laboratory, Ahmadu Bello University Teaching Hospital, Zaria, Nigeria; f Solina International Center for Research and Development, Abuja, Nigeria; g Department of Medical Laboratory Services, University of Abuja Teaching Hospital, Abuja, Nigeria; h Department of Community Medicine, University of Abuja, Abuja, Nigeria; i WHO National Polio Reference Laboratory, University of Maiduguri Teaching Hospital, Maiduguri, Nigeria

**Keywords:** HPV infection, cervical cancer, Nigeria, pooled prevalence, risk factors, HPV genotypes

## Abstract

**Background:**

There are no robust national prevalence of Human Papillomavirus (HPV) genotypes in Nigerian women despite the high burden of cervical cancer morbidity and mortality.

**The objective of study:**

This study aims to determine the pooled prevalence and risk factors of genital HPV infection in Nigeria through a systemic review protocol.

**Methods:**

Databases including PubMed, Scopus, Google Scholar and AJOL were searched between 10 April to 28 July 2020. HPV studies on Nigerian females and published from April 1999 to March 2019 were included. GRADE was used to assess the quality of evidence.

**Results:**

The pooled prevalence of cervical HPV was 20.65% (95%CI: 19.7–21.7). Genotypes 31 (70.8%), 35 (69.9%) and 16 (52.9%) were the most predominant HPV in circulation. Of the six geopolitical zones in Nigeria, northeast had the highest pooled prevalence of HPV infection (48.1%), while the least was in the north-west (6.8%). After multivariate logistic regression, duration (years) of sexual exposure (OR = 3.24, 95%CI: 1.78–9.23]), history of other malignancies (OR = 1.93, 95%CI: 1.03–2.97]), history of sexually transmitted infection (OR = 2.45, 95% CI: 1.31–3.55]), coital frequency per week (OR = 5.11, 95%CI: 3.86–14.29), the status of circumcision of the sexual partner (OR = 2.71, 95%CI: 1.62–9.05), and marital status (OR = 1.72, 95%CI: 1.16–4.72), were significant risk factors of HPV infection (*p* < 0.05). Irregular menstruation, post-coital bleeding and abdominal vaginal discharge were significantly associated with HPV infection (*p* < 0.05).

**Conclusion:**

HPV prevalence is high in Nigeria and was significantly associated with several associated risk factors. Rapid screening for high-risk HPV genotypes is recommended and multivalent HPV vaccines should be considered for women.

## Introduction

The human papillomavirus (HPV), a member of the *Papillomaviridae* family is recognized as a common and significant aetiology of sexually transmitted viral infection [1,2]. It has been estimated that approximately 5.2% of all cancers are said to be caused by HPV. Of which, cancers of the vagina, cervix, penis, vulva, anus and oropharyngeal cavity are the major categories [3]. So far, more than 200 HPV types have been recognized. Out of these, 14 are thought to be high-risk (HR) HPV types, they include types 16, 18, 31, 33, 35, 39, 45, 51, 52, 56, 58, 59, 68 and 73 [3]. Although HR HPV types account for nearly all HPV-related cancers, HPV 16 and 18 are the attributable causes of 70% of cases worldwide [4]. Globally, cervical cancer is the fourth most common cancer. The International Agency for Research on Cancer (IARC) gave a worldwide report of 569,847 new cases and 311,365 deaths due to cervical cancer in 2018 [5].

So far, there are three preventative HPV vaccines that have since been endorsed by the United States Food and Drug Administration (FDA), namely Cervarix^®^ and Gardasil^®^ [9]. These vaccines are accessible for early prophylaxis of infection with common cancer-causing HPV types. It has been reported that all the three HPV vaccines protect against HPV types 16 and 18 [6]. Moreover, Gardasil 9 can enhance protection even up to 90% of cervical cancer [6].

HPV vaccines have proven to be harmless and efficacious by offering long-term protection against HPV infections [7]. To the best of our knowledge, most of the healthcare facilities in Nigeria are yet to introduce the HPV vaccine in their national vaccination program. Thus, data on HPV genotypes, geographical distribution and risk factors among women of childbearing age are important to determine the best HPV vaccines needful for the protection against cervical cancer.

Despite the high burden of cervical cancer morbidity and mortality in Nigeria, there is no robust nationwide prevalence data on the HPV genotypes in Nigerian women. Thus, this study aims to determine the pooled prevalence and risk factors of genital HPV infection in Nigeria through systemic review. Furthermore, this study aims to promote awareness among health policymakers with regards to the establishment of robust screening programs and HPV vaccination in Nigeria.

## Methodology

Based on the guidelines highlighted in the Meta-analysis of Observational Studies in Epidemiology (MOOSE) for systematic reviews [8] and the Preferred Reporting Items for Systematic Reviews and Meta-Analyses (PRISMA) instruction for documenting meta-analysis and systematic reviews [9,10], the systematic review protocol was developed and executed. The methodology applied for this study involved cross-sectional studies.

### Search strategy

**Figure 1.**
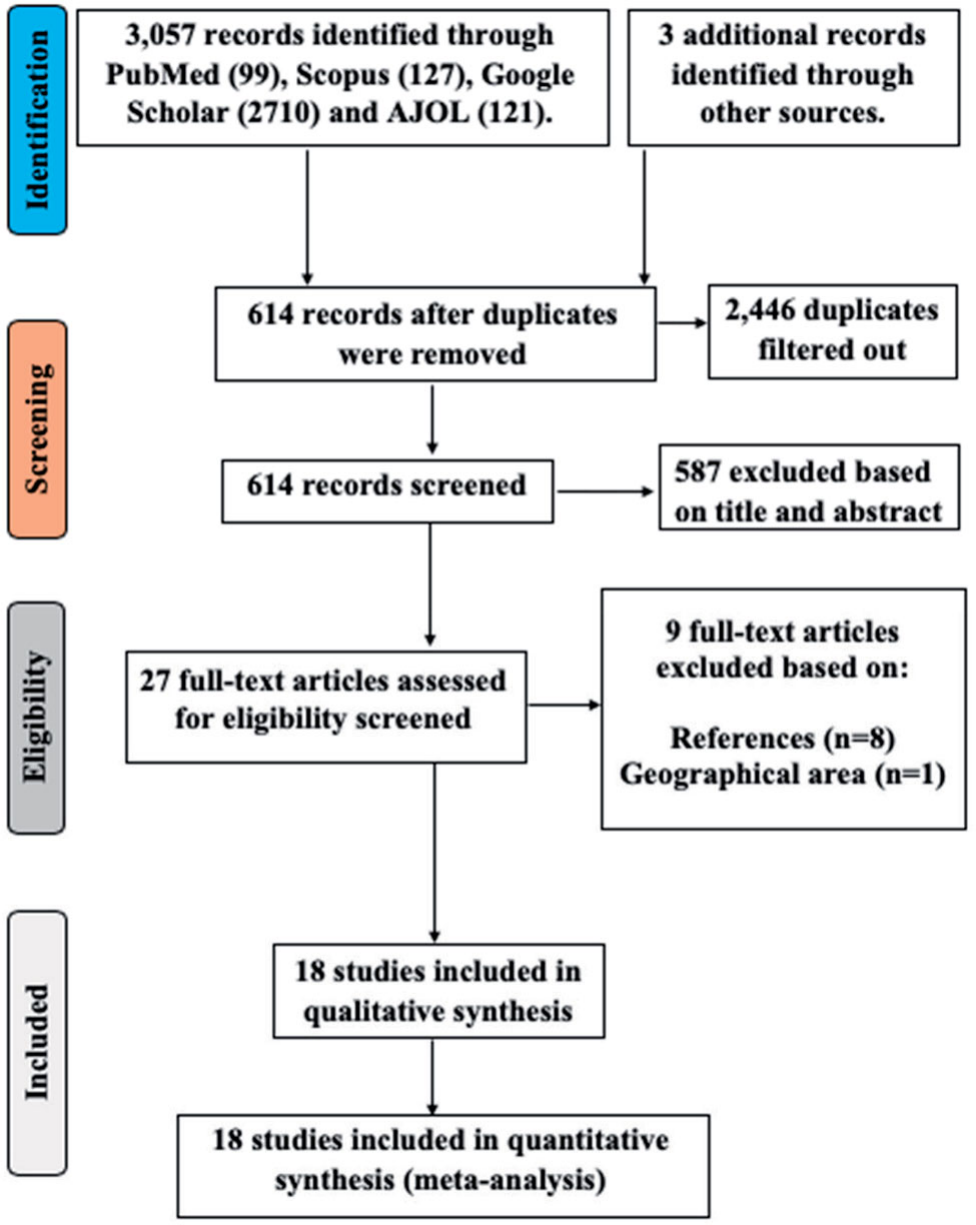
PRISMA flow diagram of search strategy for inclusion of published studies. AJOL: African Journals Online.

Using direct database search through Scopus, Web of Science, PubMed, Google Scholar and African Journal Online, the following terms and their variants were used for HPV infection research. Words such as “Human Papillomavirus,” “HPV infection,” “HPV genotypes,” “cervical cancer,” “cervical screening,” “cervical cytology,” “Pap testing” and “Nigeria.” We also used additional search terms for more abstracts on subject titles and abstracts of all eligible primary research articles. The search strategy conducted between March and April 2020 on articles published from 1st January 2000 to 28th July 2020 on the selected databases. Search outputs, numbers of included and excluded articles are presented in Figure 1. Authors independently sourced data including author’s first name, study design, publication date, research area, sampling method, criteria for inclusion of research study participants, laboratory protocol for the method of detection, geopolitical regions of participants’ enrolment, HPV genotypes, HPV severity, clinical manifestations of study participants, participants tested for HPV, the number of participants with detected with either single or mixed HPV genotype infection or both. A random-effect model was adopted to pool the prevalence of HPV infections and related sociodemographic data documented from eligible studies. Crude prevalence of HPV infection was computed based on crude numerators and denominators which were accessible from eligible studies.

### Assessment of study bias

The risk of bias in primary studies was assessed by Cochrane’s method. The sample size of every study was included as one of the criteria for determining the risk of bias, as described by the Cochrane collaboration [11]. HPV prevalence studies were evaluated in three domains, namely sampling technique, the participation level of the subjects and WNV test method. Studies were categorized as low risk of bias if they used random sampling techniques,  < 80% involvement of respondents and the use of either NAAT or NAAT and other protocol for the determination of HPV prevalence from the overall study population.

Studies that did not provide information for the three aforementioned domains were classified as having an unclear risk of bias. When a study does not have one of the domains, it was considered a moderate risk of bias. Furthermore, if two domains were not obtainable, such studies were classified to have high risk of bias. The use of random sampling technique was only applicable for studies on the general population because there will be significantly high bias in selecting participants with acute fever infection from healthcare facilities. Studies on humans were considered to have high precision if their sample sizes were greater than 100 [12].

### Studies, criteria for eligibility and quality assessment

We adopted a comprehensive approach in the identification of studies that included females of Nigerian origin either by birth, marriage or naturalization and who reside in Nigeria. This focus was made to enable us to determine the pooled prevalence of HPV infection in Nigeria. Searches were done both manually and electronically to identify studies before their listing in Endnote (version X9). Following the exclusion of duplicate citations, two independent reviewers’(I. N. A. and L. U.) examined the titles and abstracts of selected published primary manuscripts to determine the eligibility and risk of bias for each study’s full text. For a study to be eligible for our systematic review, either one or more of the criteria which include relevant data of studies that are accessible; studies that investigated HPV infection in Nigerian females residing in Nigeria alone; studies that involved cervical cytology/histology that was confirmed using exfoliated cells or fresh biopsy from the cervix; studies that involved the investigation of an abnormal cervical pap smear, HPV DNA, anti-HPV IgM, anti-HPV IgG HPV-neutralizing immunoglobulin detection; and studies with separately computed HPV genotype prevalence for every cervical lesion based on Bethesda classification. No restriction was made as regards the type of HPV assay protocol used for determining the HPV status of infected participants. HPV genotypes were determined by molecular methods (hybrid capture, reverse line-blot hybridization, polymerase chain reaction or type-specific probes) or by immunological techniques (e.g. ELISA). Published manuscripts that do not meet these criteria were excluded. Studies that lack primary data and/or well-defined methodology were also excluded from our study. Only updated and completed versions of duplicate studies were included. The search for HPV infection was conducted systematically in Nigeria both from community-based and hospital-based studies that documented HPV on human samples. When further clarifications were needed on relevant studies whose full text were not accessible, corresponding authors were contacted through e-mail. Discrepancies from data interpretation between authors were resolved based on consensus.

### Data items

For data or data subsets of the same population that were published in multiple reports [13,14], the articles were independently considered based on the different primers which were used to determine dissimilar overall HPV prevalence. While one study [13] reported the HPV genotype distribution, the other study [14] which involved the same population reported the cervical cancer screening data. One study [15] included data from Nigeria Women representing multiple states of the Federal Republic of which was data from one of the states were captured in another study [16]. To avoid duplicate representations, data from the previous study [15] which were captured in the latter study [17] were captured as one. Gage et al. [17]reported data on abnormal cytology which were not properly broken down.

### Data extraction

The main information (data) extracted from selected studies into spreadsheet for analysis included: participant features (mean or median age, standard deviation of age), study characteristics (author’s name, publication year, study location, study design, sample size, period of data collection), method of detection, PCR primers (GP5+/6+, PGMY09/11, MY095, MY115, GP-E6-3F/GP-E7-5B, GP-E7-6B, GP-E6/E7 or a combination of primers), detailed HPV investigation and genotyping protocol, number of HPV infected Nigerian females, risk factor characteristics [age at sexual initiation or debut or coitus (years), duration (years) of sexual exposure, coital frequency per week, status of circumcision of sexual partner, CD4 cell count (cells/mm^3^), level of education, employment status, marital status, parity, gravity (number of pregnancies), type of marriage, religion, contraceptive use, duration (years) of contraceptive use, chewing habit, vaginal itching, abdominal vaginal discharge, post-coital bleeding, irregular menstruation, post-menopausal age (years), post-menopausal bleeding, HIV status, HIV viral load (Copies/ml), antiretroviral drug use, herpes simplex status, direct tobacco use (direct smoking), indirect tobacco use (indirect smoking), number of sexual partners, husband’s extramarital sexual relationships, age (years) at primigravidity, family history of cervical cancer, history of other malignancies, types of other malignancies, sexually transmitted disease (STD) symptoms, STD duration (years), previous cervical screening, history of STD, previous PID/STD treatment, prevalence for high risk HPV and overall HPV prevalence were disclosed. Data were extracted both for high and low-risk HPV genotypes. Studies that documented several infections with any HPV genotype were equally extracted. Cases were categorized into grades of diagnosis based on cervical cytology/histology which included normal cytology and abnormal cytology (ASCUS: atypical squamous cells of undetermined significance; AGCUS: atypical glandular cells of undermined significance; LSIL: low-grade squamous intraepithelial lesions; HSIL: high-grade squamous intraepithelial lesions; ICC: invasive cervical cancer).

### Statistical analysis

Overall crude prevalence of HPV infection was determined as the proportion of the investigated participants that tested positive for any HPV infection expressed as a percentage [(number of HPV positive females/the total number of female participants tested) × 100]. Similarly, HPV type-specific prevalence by cervical cytology/histology was equally computed for participants who tested positive for a particular HPV genotype among all HPV-positive females who were investigated for that genotype. In HPV-specific type prevalence, just studies that investigated for a defined HPV type contributed to the analysis for that particular genotype, hence, sample sizes were dissimilar between specific HPV types that were analyzed. Medcalc software version 19.6.1 (Ostend, Belgium) was used to perform every statistical analysis. *p* values less than or equal to 0.05 at 95% confidence interval were considered statistically significant.

## Results

### Selection of studies for review

A total of 3060 references were retrieved and 27 full articles were reviewed. Data were abstracted from 18 epidemiological types of research with individual-based data attaining the criteria. The flow diagram is illustrated in Figure 1.

### Study characteristics

Data were collated from studies conducted between 2004 [18] and 2019 [13,19–22]. Except for the southeastern region of Nigeria where there were no data generated, all HPV genotyping of Nigerian participants were conducted in laboratories in North-central [15,16,20], North-east [23], North-west [15,22] and South-west [13,14,17,18,24] and South-south [25]. Two studies reported multiple infections with any specific HPV-type. A total of 6224 women from 18 Nigerian states were recruited to participate in the several studies included in the systematic review. Finally, HPV DNA testing was performed on 5390 specimens from females who had an HPV-positive test result. Their distribution by cytological category and geographical region is shown in Table 3.

Out of 5390 women tested for any HPV infection, 2935 had normal cytology; 116 were classified as ASCUS, 2 were investigated for AGSUS, 157 were diagnosed with LSIL, 61 were positive for HSIL and 60 had ICC. Also, a total of 80 females were reported as either ASCUS or LSIL, and 61 were classified as undefined abnormal cytology. Based on the classification of geographic zones of Nigeria, the South-west had the highest number of females included in the study (3812), followed by North-central (1251), South-east (445), North-west (428), North-east (208), and South-south (80) (Table 2). Ibadan in Oyo state (1004) the largest study size from two studies followed Kaduna (276), Keffi (220), Gombe (208), Ido-Ekiti (200), Okene (199), Katari (152) and Abuja (59) (Table 3).

### Cervical HPV prevalence

The crude overall HPV prevalence of each study ranged from 5.4% to 54.1%, while the prevalence of high-risk HPV of each study ranged from 5% to 46.12% (Table 1). The HPV prevalence was positively associated with study area (*p* < 0.0001, χ^2^ =61.694), cervical cells collection method (*p* = 0.0001, χ^2^ =2376.305) and HPV severity (*p* < 0.0001, χ^2^ =2267.899).

**Table 1.** Characteristics of studies included for current systematic review.

Reference	Study population; period of data collection; location	Sample size	Study design	Mean age (SD) or age range	Method of detection	Number of cases	HPV genotypes detected	Overall HPV prevalence (%)	High risk HPV prevalence (%)	Risk of bias
Akarolo-Anthony et al. [41]	General public referred for cervical screening; April–August 2012; Abuja	275	Cross-sectional	38 (8)	PCR	101	HR-16, 18, 26, 31, 33, 35, 39, 45, 51, 52, 56, 58, 59, 66, 68, 73 (MM9), 82 (MM4). LR- 6, 11, 40, 42, 53, 54, 55, 61, 62, 64, 67, 69, 70, 71, 72, 81, 83 (MM7), 84 (MM8), IS39, CP6108.	37	28.1	Low
Manga et al. [23]	General public referred for cervical screening; August–May 2013; Gombe	208	Cross sectional	39.6 (10.4)	Nested PCR (GP5+/GP6+ & PGMY09/11))	100	HR-18, 16, 31, 33, 35, 45, 56, 58, 82. LR- 38.	48.1	46.12	Moderate
Ogah et al. [19]	Participants who have not had complete or total hysterectomy recruited in November 2016	200	Cross sectional	15–50	PCR (PGMY09/11)	23	HR- 16, 18, 31, 33, 35, 39, 52, 56, 73, 81, 82. LR- 43, 44, 6, 26, 84, 70.	11.5	5	Low
Yakub et al. [20]	HIV-positive participants with no history of hysterectomy or cervical cancer recruited for onsite cervical cancer screening between August 2016-May 2017.	220	Cross sectional	30–65	Nested PCR (GP5+/GP6+/ PGMY 09/11)	119	HR-16, 18, 31, 33, 35, 39, 45, 51, 52, 56. LR- 6, 11, 40, 42, 43, 44, 66, 72, 81.	54.1	40	High
Elukunbi et al. [21]	Pregnant participants recruited between December 2014 and September 2015	93	Cross sectional	20–45	ELISA (WKEA MED SURPLUS CORP, China)	5	NA^a^	5.4	NA^a^	High
Ojiyi et al. [44]	Sexually active participants recruited between April 2004 and May 2012	445	Prospective descriptive	15–>35	Microscopy	46	NA^a^	10.3	NA^a^	Moderate
Nejo et al. [13]	Sexually active participants recruited for routine cervical cancer screening (Pap smear) between March 2014 and November 2015	295	Cross sectional	23–77	PCR (GP-E6-3F/GP-E7-5B and GP-E7-6B)	51	HR-16, 18, 31, 33, 35, 52, 58, 66. LR- 6, 42, 43, 44, 81.	17.3	14.9	Low
Kolawole et al. [24]	General public recruited for conventional Pap smear screening	200	Cross sectional	15–64	PCR (Gp5+/Gp6+)	14	NA^b^	7	NA^b^	Low
Okunade et al. [45]	General public recruited within 6 months for routine cytological evaluation and pelvic examination with the exclusion of virgins, pregnant women, those who have undergone hysterectomy, individuals with cervical lesions and the mentally as well as physically challenged.	200	Cross sectional	36.1 (7.4)	PCR	73	HR- 16, 31, 35.	36.5	36.5	Low
Nejo et al. [14]	Sexually active participants recruited for routine cervical cancer screening (Pap smear) between March 2014 and November 2015	295	Cross sectional	23–77	PCR (GP-E6/E7 /PGMY09/11)	55	NA^b^	18.6	NA^b^	Low
Pimentel et al. [15]	Recruitment of the general public of those with uterus, are not pregnant, no history of cervical dysplasia or cancer from 3 geographical regions between 2004 and 2008	410	Cross sectional	36.2 (10.7)	HC-II	64	HR-16, 18, 31, 33, 35, 39, 45, 51, 52, 56, 58, 59, 68.	16.0	16.0	Low
Thomas et al. [18]	Sexually active individuals recruited for routine cervical cancer screening (Pap smear) between April and May 1999	932	Population-based	15–>65	PCR (GP5+/6+) /EIA/reverse line blot hybridization	245	HR-16, 18, 26, 31, 33, 35, 39, 45, 51, 52, 53, 56, 58, 59, 66, 68, 73, and 82. LR-6, 11, 34, 40, 42, 43, 54, 55, 70, 72, 81, 83, 84, CP6108.	26.3	19.7	Low
Modibbo et al. [46]	Sexually active participants recruited for cervical cancer screening between February and May 2014	298	Community based	30–65	PCR (GP5+/6+)	29	HR-35, 52, 66, 18, 56, 58, 51, 39, 16, 33, 45.	10	10	Low
Kennedy et al. [25]	General public recruited for cancer screening between August and December 2014	80	Cross sectional	39 (5)	PCR	8	HR- 16, 18, 31 and 35	10	10	Moderate
Magaji et al. [22]	Sexually active participants recruited for cervical screening in 2015	276	Hospital based and cross sectional	37–56	ELISA & PCR (MY095/ MY115)	20	HR-16, 18, 31, 45	7.2	7.2	Low
Gage et al. [17]	Non-virgin participants enrolled for cervical evaluation	1282	Population-based & cross sectional	15–>70	PCR (MY095/MY115)	188	HR-16, 18, 31, 33, 35, 39, 45, 51, 52, 56, 58, 59 and 68	14.7	14.7	Low
Ezechi et al. [47]	Recruitment of HIV positive and negative participants for cervical screening	515	Cross sectional	18–81	PCR	101	HR-16, 18, 31, 33, 35, 39, 45, 51, 52, 56, 58, 59, 68.	19.6	19.6	Low
Schnatz et al. [16]	Recruitment of participants for cervical examination who were not pregnant, not with any known history of cervical dysplasia or cancer, with uterus and whose Pap smear was technically able to be analyzed	199	Cross sectional	33.2 (8.6)	PCR	43	HR- 16, 18, 31, 33, 35, 39, 45, 51, 52, 56, 58, & 68. LR- 6, 11, 42, 43, & 44.	21.6	16.6	Low

^a^NA: not applicable.

^b^NA: not available.

**Table 2.** Pooled prevalence of HPV infection in Nigeria.

Features	Categories	Number of studies	Number of participants	Number of HPV cases (%)	*p*-value	Chi-square
Study area	Rural	2	1481	231 (15.6)	˂0.0001	61.694
	Urban	12	3340	785 (23.5)		
	Semi-urban	1	298	29 (9.7)		
	Rural & urban	3	1105	207 (18.7)		
Geo-political zone	North-east	1	208	100 (48.1)	˂0.0001	209.656
	North-west	2^a^	428	29 (6.8)		
	North-central	6^a^	1251	324 (25.9)		
	South-west	8	3812	732 (19.2)		
	South-east	1	445	46 (10.3)		
	South-south	1	80	8 (10.0)		
Study design	Cross sectional	13	3190	757 (23.7)	˂0.0001	67.45
	Prospective descriptive	1	445	46 (10.3)		
	Population-based	1	932	245 (26.3)		
	Community based	1	298	29 (9.7)		
	Hospital-based and cross-sectional	1	276	20 (7.2)		
	Population-based & cross sectional	1	1282	188 (14.7)		
Cervical cells method of collection	Endocervical /flocked Swab	3	793	153 (19.3)	0.0001	2376.305
	Cytobrush/cervical brush	11	4597	1012 (22.0)		
	Undisclosed	1	200	14 (7.0)		
Laboratory protocol for HPV detection	Microscopy	1	445	46 (10.3)	˂0.0001	1229.2
	PCR	13	4267	905 (21.2)		
	ELISA	1	93	5 (5.4)		
	HC-II	1	410	64 (15.6)		
	ELISA & PCR	1	276	20 (7.2)		
	Reverse line blot hybridization, EIA and PCR	1	932	245 (26.3)		
Primer set	GP5^+^/6^+^	3	1430	288 (20.1)		
	PGMY09/11	1	200	23 (11.5)		
	MY095 & MY115	2	1558	138 (8.9)		
	GP5+/GP6+ & PGMY09/11	2	428	219 (51.2)		
	GP-E6-3F/GP-E7-5B & GP-E7-6B	1	295	51 (17.3)		
	GP-E6/E7 & PGMY09/11	1	295	55 (18.6)		
	Undisclosed	5	1269	326 (25.7)		
HPV severity	High-risk HPV	7	3061	483 (15.8)	˂0.0001	2267.899
	Low-risk HPV	0	0	0 (0.0)		
	High- & low-risk HPV	7	2329	682 (29.3)		

PCR: polymerase chain reaction; HC-II: hybrid capture assay 2.

^a^Included a study with study areas from two different geographical regions.

**Table 3.** Regional specific distribution of studies, study size and prevalence of HPV by cervical disease grade and region.

Nigerian geographical region (no. of studies)	Location [reference]	Total (no. of studie*s* = 9)		Normal cytology (no. of studie*s* = 3)		ASCUS (no. of studie*s* = 3)		AGCUS (no. of studie*s* = 3)		LSIL (no. of studie*s* = 3)		HSIL (no. of studie*s* = 3)		ICC (no. of studie*s* = 3)	
		Tested N	HPV (+), n	Tested N	HPV (+), n (%)	Tested N	HPV (+), n (%)	Tested N	HPV (+), n (%)	Tested N	HPV (+), n (%)	Tested N	HPV (+), n (%)	Tested N	HPV (+), n (%)
North-central	Keffi [4]	220	119	6	NA	NA	NA	NA	NA	NA	NA	NA	NA	54	NA
	Abuja [11]	59	9	53	3 (5.7)	1	NA	0	0 (0)	3	NA	2	NA	0	0 (0)
	Okene [18]	199	43	186	35 (18.8)	9	5 (55.6)	1	0 (0)	2	1 (50)	1	1 (100)	0	0 (0)
North-east	Gombe [2]	208	100	126	65 (51.6)	NA	NA	NA	NA	NA	NA	3	3 (100)	3	3 (100)
North-west	Katari [11]	152	22	143	13 (9.1)	2	NA	0	0 (0)	6	NA	1	NA	0	0 (0)
	Kaduna [15]	276	20	255	6 (2.4)	2	1 (50)	0	0 (0)	13	11 (84.6)	2	2 (100)	0	0 (0)
South-west	Ido-Ekiti [8]	200	14	186	0 (0)	3	3 (100)	0	0 (0)	7	7 (100)	4	4 (100)	0	0 (0)
	Ibadan [10,12]	1004^d^	253	905	215 (23.8)	19	0 (0)^e^	1	0 (0)^e^	46	1 (11.1)^e^	16	0 (0)^e^	3	3 (100)
	Irun [16]	1282^c^	188	1075	113 (10.5)	80^b^	18 (NA)	0	0	80^b^	23 (NA)	32	21 (65.6)	0	0 (0)
Overall (10)	–	3600^d,c^	768	2935	450 (15.3)^d,e^	116^b,e^	27 (23.3)^e^	2	0 (0)^e^	157^b,e^	43 (27.4)^e^	61^e^	31 (50.8)^e^	60	6 (10.0)^e^
95% CI					14.1–16.7^d,e^		15.9–32.0^e^				20.6–35.1^e^		37.7–63.9^e^		8.3–28.5^e^

ASCUS: atypical squamous cells of undetermined significance; AGCUS: atypical glandular cells of undermined significance; LSIL: low-grade squamous intraepithelial lesions; HSIL: high-grade squamous intraepithelial lesions; ICC: invasive cervical cancer; NA: not assessable.

^a^Includes 4 inadequate samples.

^b^Represent data for ASCUS, AGCUS & LSIL.

^c^Cytological report for 95 participants are not available.

^d^Cytological report for 14 participants are missing.

^e^Contains non-accessible data.

### HPV genotype distribution

**Figure 2.**
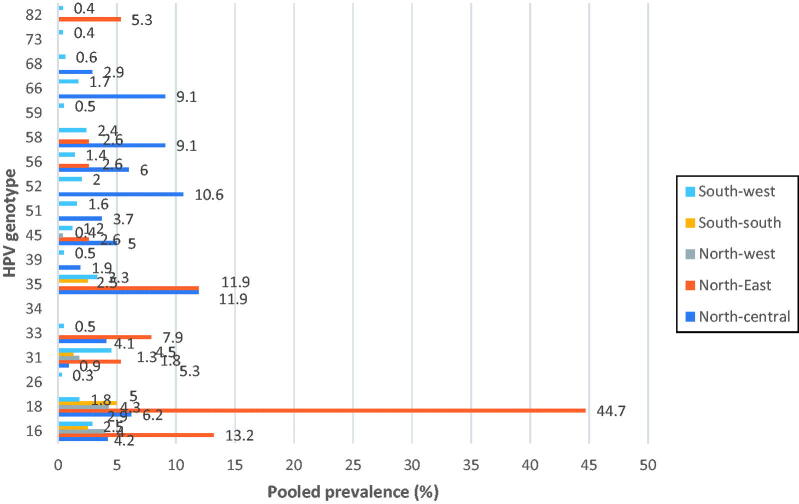
Prevalence of the most common high-risk human papillomavirus (HPV) genotypes by geographical regions among Nigerian women. *NB*: There were no data representations from the south-eastern region.

As shown in Figure 2, based on the studies that documented detailed breakdown of HPV genotype-specific distribution, the 10 most frequent high-risk HPV genotypes among Nigerian women in South-west were HPV 31 (4.5%), 35 (3.3%), 16 (2.9%), 58 (2.4%), 52 (2%), 18 (1.8%), 66 (1.7%), 51 (1.6%), 56 (1.4%) and 45 (1.2%) in descending order. On the other hand, the most frequent high-risk HPV genotypes among Nigerian women in the South-south are HPV 18 (5%), 16 (2.5%), 35 (2.5%) and 31 (1.3%) in descending order. In North-western Nigeria, the most frequent high-risk HPV genotypes among females were HPV 18 (4.3%), 16 (4%), 31 (1.8%) and 45 (0.4%) in descending order. The HPV genotype distribution in the North-eastern part of Nigeria were HPV 18 (44.7%), 16 (13.2%), 35 (11.9%), 33 (7.9%), 82 (5.3%), 31 (5.3%), 45 (2.6%), 56 (2.6%) and 58 (2.6%) in descending order. The North-central region of Nigeria had the most frequent high-risk HPV genotypes among females which include HPV 18 (4.3%), 16 (4%), 31 (1.3%) and 45 (0.4%) in descending order. Overall HPV 18 was among the most prevalent genotypes, ranking first both in North-east, Northwest and South-south, but fifth in North-central and sixth in South-west. There was no study representing females from South-eastern Nigeria that met the inclusion criteria. The North-central and South-west had HPV 35 and HPV 31 as their respective most prevalent genotypes. There was a strong positive association between the genotype distribution of HPV-positive cases and the geo-political zones of Nigeria (χ^2^ = 209.656, *p* < 0.0001) (Table 2).

### Outputs analysis based on Bethesda classification

The analysis of the prevalence of HPV specific types based on the category of lesion according to the Bethesda classification of females with normal cytology and cervical neoplastic lesions (ASCUS, AGCUS, LSIL, HSIL and ICC) are presented in Table 4.

**Table 4.** Prevalence of HPV by Bethesda classification by cytology.

	Normal cytology		ASCUS		LSIL		HSIL		ICC	
HPV types	Sample size, N	Cases (studies)	Sample size, N	Cases (studies)	Sample size, N	Cases (studies)	Sample size, N	Cases (studies)	Sample size, N	Cases (studies)
HIGH-RISK										
16	33	16 (3)^a^	5^b,c^	3 (3)^a^	3^c^	3 (3)^a,c^	10	7 (3)^a^	0^a^	0 (3)^a^
18	45	13 (3)^a^	2^b,c^	1 (3)^a^	1^c^	1 (3)^a,c^	2	2 (3)^a^	3	0 (3)^a^
26	0	0 (1)^a^	1^b^	0 (1)^a^	0	0 (1)^a^	0	0 (1)	0	0 (1)^a^
31	32	21 (3)^a^	10^b,c^	6 (3)^a^	6^c^	6 (3)^a,c^	3	3 (3)^a^	0	0 (3)^a^
33	8	2 (3)^a^	0	0 (3)^a,c^	0	0 (3)^a^	1	1 (3)^a^	0	0 (3)^a^
34	0	0 (0)	0	0 (0)	0	0 (0)	0	0 (0)	0	0 (0)
35	31	16 (3)^a^	8^b^	7 (3)^a,c^	7^c^	7 (3)^a,c^	6	6 (3)^a^	0	0 (3)^a^
39	5	3 (2)^a^	2	2 (1)^a,c^	2^c^	2 (2)^a,c^	0	0 (2)^a^	0	0 (2)^a^
45	16	4 (3)^a^	1^b^	0 (3)^a^	0	0 (3)^a^	2	2 (3)^a^	0	0 (3)^a^
51	15	9 (2)^a^	10^b^	9 (2)^a^	9	9 (2)^a,c^	1	1 (2)^a^	0	0 (2)^a^
52	23	20 (2)^a^	9^b^	7 (2)^a^	7^c^	7 (2)^a,c^	2	2 (2)^a^	0	0 (2)^a^
53	1	0 (1)^a^	0	0 (1)^a^	0	0 (1)^a^	0	0 (1)^a^	0	0 (1)^a^
56	15	6 (3)^a^	5^b^	3 (3)^a,c^	3	3 (3)^a,c^	0	0 (3)^a^	0	0 (3)^a^
58	28	18 (3)^a^	8^b^	7 (3)^a,c^	7^c^	7 (3)^a,c^	1	1 (3)^a^	0	0 (3)^a^
59	7	4 (2)^a^	2	2 (2)^a,c^	2	2 (2)^a,c^	0	0 (2)^a^	0	0 (2)^a^
66	12	0 (1)^a^	0	0 (1)^a^	0	0 (1)^a^	0	0 (1)^a^	0	0 (1)^a^
68	8	8 (2)^a^	2	2 (2)^a,c^	2	2 (2)^a,c^	0	0 (2)^a^	0	0 (2)^a^
73	0	0 (1)^a^	0	0 (1)^a^	0	0 (1)^a^	0	0 (1)^a^	0	0 (1)^a^
82	7	0 (2)^a^	1^b^	0 (2)^a^	0	0 (2)^a^	0	0 (2)^a^	0	0 (2)^a^
6	2	0 (1)^a^	0	0 (1)^a^	0	0 (1)^a^	0	0 (1)^a^	0	0 (1)^a^
11	0	0 (1)	0	0 (1)	0	0 (1)	0	0 (1)	0	0 (1)
34	0	0 (1)	0	0 (1)	0	0 (1)	0	0 (1)	0	0 (1)
38	3	0 (1)^a^	0	0 (1)^a^	0	0 (1)^a^	0	0 (1)^a^	0	0 (1)^a^
40	3	0 (1)^a^	1^b^	0 (1)^a^	0	0 (1)^a^	0	0 (1)^a^	0	0 (1)^a^
42	20	0 (1)^a^	0	0 (1)^a^	0	0 (1)^a^	0	0 (1)^a^	0	0 (1)^a^
43	1	0 (1)^a^	0	0 (1)^a^	0	0 (1)^a^	0	0 (1)^a^	0	0 (1)^a^
53	0	0 (0)	0	0 (0)	0	0 (0)	0	0 (0)	0	0 (0)
54	0	0 (1)	0	0 (1)	0	0 (1)	0	0 (1)	0	0 (1)
55	0	0 (1)	0	0 (1)	0	0 (1)	0	0 (1)	0	0 (1)
61	0	0 (0)	0	0 (0)	0	0 (0)	0	0 (0)	0	0 (0)
62	0	0 (0)	0	0 (0)	0	0 (0)	0	0 (0)	0	0 (0)
64	0	0 (0)	0	0 (0)	0	0 (0)	0	0 (0)	0	0 (0)
67	0	0 (0)	0	0 (0)	0	0 (0)	0	0 (0)	0	0 (0)
69	0	0 (0)	0	0 (0)	0	0 (0)	0	0 (0)	0	0 (0)
70	3	0 (1)^a^	1^b^	0 (1)^a^	0	0 (1)	0	0 (1)	0	0 (1)
71	0	0 (0)	0	0 (0)	0	0 (0)	0	0 (0)	0	0 (0)
72	4	0 (1)^a^	2^b^	0 (1)	0	0 (1)	0	0 (1)	0	0 (1)
81	9	0 (1)^a^	1^b^	0 (1)	0	0 (1)	0	0 (1)	0	0 (1)
83	4	0 (1)^a^	0	0 (1)	0	0 (1)	0	0 (1)	0	0 (1)
84	2	0 (1)^a^	0	0 (1)	0	0 (1)	0	0 (1)	0	0 (1)
KC5	0	0 (0)	0	0 (0)	0	0 (0)	0	0 (0)	0	0 (0)
IS39	0	0 (0)	0	0 (0)	0	0 (0)	0	0 (0)	0	0 (0)
CP6108	0	0 (1)	0	0 (1)	0	0 (1)	0	0 (1)	0	0 (1)
Mult	183	0 (2)^a^	39^b^	0 (2)^a^	39^b^	0 (2)^a^	39^b^	0 (2)^a^	39^b^	0 (2)^a^
Any	0	0 (0)	0	0 (0)	0	0 (0)	0	0 (0)	0	0 (0)

Mult: multiple HPV genotypes.

Any: any other non-high-risk HPV genotypes.

^a^Contains non-accessible data.

^b^Include data for undefined abnormal cytology.

^c^Include data for ASCUS & LSIL.

### Prevalence of HPV genotypes in females with normal cervical cytology

The systematic review included 3 studies testing for cervical HPV infection in 2,935 females with normal cytology. Most of the females tested for HPV infections were from South-western Nigeria (*n* = 2166), followed by North-western Nigeria (*n* = 398), North-central Nigeria (*n* = 245) and North-east (*n* = 126) (Table 3). HPV18, 16, 31, 35, 58, 52, 45, 51, 56 and 66 were the ten most common genotypes in females with normal cervical cytology in descending order (Table 4).

Overall, six studies stratified eligible females screened for HPV infection according to age ranges. Analysis of these studies revealed that the age range 60–69 years had the highest overall HPV prevalence at 73.2%, closely followed by the age group 30–39 years with 64.3%, ≥70 years with 63.6%, 50–59 years with 62.9%, 15–29 years with 53.1% and 40–49 years with 46.8% (Table 5).

**Table 5.** Overall prevalence of any HPV based on publication year (Y), primers (P) and age groups (A).

Features	Category	Normal cytology		ASCUS		LSIL		HSIL		ICC	
		Cases/ sample size (studies)	Prevalence (%)	Cases/ Sample size (studies)	Prevalence (%)	Cases/ sample size (studies)	Prevalence (%)	Cases/ sample size (studies)	Prevalence (%)	Cases/ sample size (studies)	Prevalence (%)
Year of publication	2004	209/324 (1)	64.5	36/61^a^ (1)	59.0	36/61 (1)^a^	59.0	36/61^a^	59.0	36/61^a^	59.0
	2008	35/43 (1)	81.4	6/9 (1)	66.7	1/2 (1)	50.0	1/1 (1)	100	0/0 (1)	0.00
	2012	113/1075 (1)	10.5	41/80^b^ (1)	51.3	41/80^b^ (1)	51.3	21/32 (1)	65.6	0/0 (1)	0.00
	2016	0/186 (1)	0.00	3/3 (1)	100	7/7 (1)	100	4/4 (1)	100	0/0 (1)	0.00
	2018	6/61 (1)	9.8	0/0 (1)	0.00	1/9 (1)	11.1	0/1 (1)	0.00	1/1 (1)	100
	2019	6/255 (1)	23.5	1/2 (1)	50.0	11/13 (1)	8.5	2/2 (1)	100	0/0 (1)	0.00
Total		369/1944 (6)	18.9	87/155 (6)^a,b^	56.1	97/172 (6)^a,b^	56.4	64/101 (6)^a^	63.3	37/62 (5)^a^	59.7
Primer	GP5^+^/6^+^	209/324 (1)	64.5	36/61 (1)^a^	59.0	36/61 (1)^a^	59.0	36/61 (1)^a^	59.0	36/61 (1)^a^	59.0
	MY095 & MY115	6/255 (1)	2.4	1/2 (1)	50.0	11/13 (1)	84.6	2/2 (1)	100	0/0 (1)	0.00
	GP-E6/E7 & PGMY09/11	113/1075 (1)	10.5	41/80^b^ (1)	51.3	41/80^b^ (1)	51.3	21/32 (1)	65.6	0/0 (1)	0.00
	Undisclosed	35/43 (1)	81.4	6/9 (1)	66.7	1/2 (1)	50.0	1/1 (1)	100	0/0 (1)	0.00
Total		363/1697 (4)	21.4	84/152 (4)^a^	55.3	89/156 (4)	57.1	60/96 (4)	62.5	36/61 (4)	59.0
Age range	15–29	26/49 (1)	53.1	7/49 (1)	14.3	13/49 (1)	26.5	2/49 (1)	4.1	0/49 (1)	0.00
	30–39	18/28 (1)	64.3	3/28 (1)	10.7	2/28 (1)	7.1	3/28 (1)	10.7	0/28 (1)	0.00
	40–49	15/32 (1)	46.8	4/32 (1)	12.5	4/32 (1)	12.5	6/32 (1)	18.8	0/32 (1)	0.00
	50–59	17/27 (1)	62.9	1/27 (1)	3.7	1/27 (1)	3.7	4/27 (1)	14.8	0/27 (1)	0.00
	60–69	30/41 (1)	73.2	2/41 (1)	4.9	3/41 (1)	7.3	4/41 (1)	9.8	0/41 (1)	0.00
	≥70	7/11 (1)	63.6	1/11 (1)	9.1	0/11 (1)	0.00	2/11 (1)	18.2	0/11 (1)	0.00
Total		113/188 (6)	60.1	18/188 (6)	9.6	23/188 (6)	12.2	21/188 (6)	11.2	0/188 (6)	0.00

ASCUS: atypical squamous cells of undetermined significance; AGCUS: atypical glandular cells of undermined significance; LSIL: low-grade squamous intraepithelial lesions; HSIL: high-grade squamous intraepithelial lesions; ICC: invasive cervical cancer; NA: not available.

^a^Include data for undefined abnormal cytology.

^b^Include data for ASCUS & LSIL.

**Table 6.** Pooled sociodemographic risk factors of HPV infection in Nigeria.

Variables	Categories	No. of pooled participants (no. of studies)	No. of pooled HPV-positive cases (%)	OR (95% CI)	*p* value
Age (years)	< 30	1081 (11)	231 (21.4)	0.78 (0.45–6.98)	0.218
	30–40	1407 (11)	341 (24.2)		
	>40	1634 (11)	373 (22.8)		
Age at sexual initiation or debut or coitus (years)	< 18	977 (8)	239 (24.5)	0.15 (0.023–2.91)	0.421
	≥18	1872 (8)	405 (21.6)		
Duration (years) of sexual exposure	≤10	262 (1)	21 (8.0)	3.24 (1.78–9.23)	0.018
	11–20	137 (1)	14 (10.2)		
	≥21	46 (1)	11 (23.9)		
Coital frequency per week	1–2	312 (1)	23 (7.4)	5.11 (3.86–14.29)	0.004
	3–4	118 (1)	17 (14.4)		
	≥5	15 (1)	6 (40.0)		
Status of circumcision of sexual partner	Yes	85 (1)	15 (17.6)	2.71 (1.62–9.05)	0.031
	No	115 (1)	8 (7.0)		
CD4 cell count (cells/mm^3^)	<200	22 (1)	12 (54.5)		
	200–499	86 (1)	24 (27.9)	1.91 (0.75–26.99)	0.061
	>500	112 (1)	18 (16.1)		
Level of education	Primary	773 (8)	179 (23.2)	1.37 (0.34–5.23)	0.731
	Secondary	797 (8)	152 (19.1)		
	Tertiary	567 (8)	134 (23.6)		
	Others	15 (8)	12 (80.0)		
	No formal education	595 (8)	154 (25.9)		
Employment status	Employed	970 (5)	152 (15.7)	5.31 (2.91–11.45)	0.025
	Unemployed	150 (5)	62 (41.3)		
Marital status	Married	2194 (5)	475 (21.6)	1.71 (1.16–4.72)	0.047
	Single	200 (5)	72 (36.0)		
Parity	Nulliparous (no birth)	364 (7)	58 (15.9)	1.08 (0.39–5.19)	0.077
	Primiparous (1 birth)	453 (7)	95 (21.0)		
	Multiparous (≥2 births)	1011 (7)	235 (23.2)		
Gravity (number of pregnancies)	None	57 (1)	22 (38.6)		
	Single	134 (1)	39 (29.1)	0.67 (0.13–8.11)	0.065
	Multiple	720 (1)	181 (25.1)		
Type of marriage	Monogamy	552 (4)	104 (18.8)	0.18 (0.08–6.12)	0.138
	Polygamy	311 (4)	61 (19.6)		
Religion	Islam	134 (2)	58 (43.3)	0.29 (0.13 − 2.11)	0.058
	Christianity	519 (2)	88 (17.0)		
Contraceptive use	Yes	867 (6)	264 (30.4)	0.95 (0.84–4.18)	0.061
	No	3,936 (6)	968 (24.6)		
Duration (years) of contraceptive use	<1	62 (1)	3 (4.8)		
	1–5	38 (1)	15 (39.5)	1.22 (1.08–5.19)	0.049
	≥6	26 (1)	18 (69.2)		
Chewing habit	Yes	331 (1)	98 (29.6)	0.29 (0.07–3.19)	0.613
	No	600 (1)	147 (24.5)		
Vaginal itching	Yes	269 (1)	30 (11.2)	0.58 (0.18–3.8)	0.328
	No	176 (1)	16 (9.1)		
Abdominal vaginal discharge	Yes	269 (1)	38 (14.1)	1.89 (1.01–5.61)	0.044
	No	176 (1)	8 (4.5)		
Post-coital bleeding	Yes	8 (1)	3 (37.5)	3.51 (1.06–7.08)	0.033
	No	437 (1)	43 (9.8)		
Irregular menstruation	Yes	13 (1)	6 (46.2)	3.79 (1.23–6.88)	0.028
	No	432 (1)	40 (9.3)		
Post-menopausal age (years)	1-5	48 (1)	8 (16.7)	1.19 (0.39–7.1)	0.063
	6-10	23 (1)	3 (13.0)		
	≥11	10 (1)	3 (30.0)		
Post-menopausal bleeding	Yes	3 (1)	1 (33.3)	0.66 (0.084–4.21)	0.322
	No	442 (1)	45 (10.2)		
HIV status	Positive	258 (3)	70 (27.1)		
	Negative	576 (3)	158 (27.4)	0.13 (0.09–3.15)	0.173
HIV viral load (copies/ml)	<1000	136 (1)	19 (14.0)		
	1000–9999	26 (1)	6 (23.1)	5.78 (1.92–7.82)	0.042
	>10,000	58 (1)	29 (50.0)		
Antiretroviral drug use	Not on drugs	61 (1)	24 (39.3)	3.15 (1.07–8.1)	0.006
	On drugs	159 (1)	30 (18.9)		
Herpes simplex status	Yes	547 (1)	163 (29.8)		
	No	345 (1)	74 (21.4)	0.87 (0.209–5.11)	0.076
Direct tobacco use (direct smoking)	Yes	5 (2)	1 (20.0)		
	No	370 (2)	62 (16.8)	0.33 (0.06–3.81)	0.122
Indirect tobacco use (indirect smoking)	Yes	5 (1)	3 (60.0)		
	No	290 (1)	52 (17.9)	4.49 (2.13–11.89)	0.0022
Number of sexual partners	Single	1557 (10)	327 (21.0)		
	Multiple	1774 (10)	396 (22.3)	0.52 (0.043–3.19)	0.0732
Husband’s extramarital sexual relationships	No	158 (2)	32 (20.3)		
	Uncertain	162 (2)	41 (25.3)	0.29 (0.032–3.17)	0.089
	Yes	692 (2)	180 (26.0)		
Age (years) at primigravidity	<18	172 (3)	63 (36.6)		
	18-28	759 (3)	232 (30.6)	0.83 (0.65–6.77)	0.091
	>28	330 (3)	96 (29.1)		
Family history of cervical cancer	No	181 (1)	85 (47.0)	0.43 (0.28–2.11)	0.181
	Yes	25 (1)	14 (56.0)		
History of other malignancies	No	179 (1)	80 (44.7)	1.93 (1.03–2.97)	0.037
	Yes	26 (1)	19 (73.1)		
Types of other malignancies	Gynecological	23 (1)	17 (73.9)	2.41 (0.86–6.98)	0.056
	Others	2 (1)	1 (50.0)		
STD symptoms	Yes	121 (1)	25 (20.7)	1.31 (0.78–4.22)	0.231
	No	174 (1)	30 (17.2)		
STD duration (years)	1-10	111 (1)	23 (20.7)	0.23 (0.087–2.11)	0.134
	11–20	5 (1)	1 (20.0)		
	>20	5 (1)	1 (20.0)		
Previous cervical screening	Yes	72 (1)	8 (11.1)	0.48 (0.14–3.46)	0.0776
	No	223 (1)	47 (21.1)		
History of STD	Yes	8 (1)	6 (75.0)	2.45 (1.31–3.55)	0.023
	No	72 (1)	2 (2.8)		
Previous PID/STD treatment	Yes	396 (3)	58 (14.6)	0.71 (0.41–4.12)	0.245
	No	544 (3)	116 (21.3)		

PID: pelvic inflammatory disease; STD: sexually transmitted disease; HIV: human immunodeficiency virus; HPV: human papillomavirus.

### Prevalence of HPV genotypes in females with atypical squamous cells of undetermined significance (ASCUS) and low-grade squamous intraepithelial lesions (LSIL)

Our systematic review included three studies testing for HPV infection in 116 females diagnosed with ASCUS. Most of the females that tested for HPV infections were from South-west (*n* = 102), followed by North-central (*n* = 10) and North-west (*n* = 4) (Table 3).

HPV 31, 51, 52, 35, 58, 16, 56, 18, 39 and 59 were the ten most common genotypes in females with ASCUS in descending order (Table 4).

Analysis of studies with ASCUS cases revealed that the age range 15–29 years had the highest overall HPV prevalence at 14.3%, closely followed by the age group 40–49 years with 12.5%, 30–39 years with 10.7%, ≥70 years with 9.1%, 60–69 years with 4.9% and 50–59 years with 3.7% (Table 5).

For LSIL, data from a total of 157 females from 3 studies were abstracted. Most of the females that tested for HPV infections were from South-west (*n* = 133), followed by North-west (*n* = 19) and North-central (*n* = 5) regions of Nigeria (Table 3). HPV 51, 35, 52, 58, 31, 16, 56, 39, 59 and 68 were the 10 most common genotypes in females with LSIL in descending order (Table 4).

Analysis of studies with LSIL cases revealed that the age range 15–29 years had the highest overall HPV prevalence at 26.5%, closely followed by the age group 40–49 years with 12.5%, 60–69 years with 7.3%, 30–39 years with 7.1% (95% CI: XX, 40–49 years with 3.7% and ≥70 years with 0.0% (Table 5).

### Prevalence of HPV genotypes in women with atypical glandular cells of undermined significance (AGCUS)

In our systematic review, three studies comprising of a total of two AGCUS cases were included in the analysis (Table 6). One participant each from North-central and South-west regions of Nigeria were enrolled in our study selection criteria (Table 3).

### Prevalence of HPV genotypes in women with high-grade squamous intraepithelial lesions (HSIL)

A total of 61 cases from 3 studies were included in the HSIL systematic review analysis. A majority of HSIL cases were from South-western region (*n* = 52), followed co-equally by North-central, North-eastern and North-western regions of Nigeria with 3 participants each from of these regions (Table 3).

HPV 16, 35, 31, 18, 45, 52, 33, 51 and 58 were the most common genotypes in females with HSIL in descending order (Table 4).

Analysis of studies with HSIL cases revealed that the age range 15–29 years had the highest overall HPV prevalence at 26.5%, closely followed by the age group 40–49 years with 12.5%, 60–69 years with 7.3%, 30–39 years with 7.1%, 40–49 years with 3.7% and ≥70 years with 0.0% (Table 5).

### Prevalence of HPV genotypes in women with invasive cervical cancer (ICC)

A total of 60 cases from 3 studies were included in the ICC systematic review analysis (Table 3). Most of the ICC cases were from the North-central region (*n* = 54), followed co-equally by the North-eastern and South-western regions of Nigeria with 3 participants each from of these regions (Table 3). HPV 16, 35, 31, 18, 45, 52, 33, 51 and 58 were the most common genotypes in females with ICC in descending order (Table 4).

## Discussion

Cervical cancer is considered as the major cause of maternal death with consequential loss of productive life as a result of cancer excruciation and healthcare cost in Africa [26]. The burden of carcinoma of the cervix has been mainly observed in women within the age range of 15–44 years of age [27]. Fourteen high-risk HPV genotypes have been incriminated in the development of the neoplasm of the cervix for several years now [27]. Unlike other continents and despite the large number of HPV-associated risk factors which can predispose women to cervical cancer development and poor prognosis [28], adequate records of prevalence and distribution of HPV genotypes are grossly insufficient in Africa. To prevent cervical cancer-associated mortality, several advanced countries have adopted and continuously reviewed their Papanicolaou screening techniques and vaccine programs [29,30]. The adoption of these efficient preventive approaches has more or less limited their applicability in Nigeria due to several socio-economic and logistical challenges.

After bivariate logistic regression, history of sexual exposure, other malignancies, sexually transmitted infection, coital frequency (per week) and circumcision status of sexual partner marital status were significant risk factors associated with HPV infection. Also, irregular menstruation, post-coital bleeding and abdominal vaginal discharge were significantly associated with HPV infection observed in our study. These findings agree with those of Soohoo et al. [31].The increased risk of contacting HPV infection among women could due to these large number of factors.

Data on HPV infections, genotypes, diagnosis, geographical distribution and risk factors among women of childbearing age are important to determine the best HPV vaccines to be adopted in protection against cervical cancer and the identification of the ideal laboratory test for diagnosis of high-risk women.

To the best of our knowledge, our study is the first systematic review in Nigeria to determine HPV genotype distribution, pooled prevalence and risk factors in women by geographical zones, lesion type, age group, publication year and PCR primers for genotyping. Eighteen studies matched the inclusion criteria for this systematic review.

Each of the six geopolitical zones in Nigeria was fairly represented. Overall, the prevalence of cervical HPV infection in Nigeria was higher when compared with other regions in Africa; our study revealed the prevalence rates in women with normal cervical cytology (15.3%), ASCUS (23.3%), LSIL (27.4%), HSIL (50.8%) and ICC (10.0%). Furthermore, this study revealed that by pooling several studies examining HPV infection among women with normal cervical cytology, of the six geopolitical zones in Nigeria, North-east had the highest pooled prevalence of HPV infection (48.1%), followed by Northcentral (25.9%), Southwest (19.2%), Southeast (10.3%), South-south (10.0%), while the least was in the North-west (6.8%). However, the prevalence of any HPV infection among women with normal cytology was low (15.3%) compared to other regions which ranged between 27.8% and 57.3%. The observed variations in the HPV prevalence rates may be suggestive of the influence of geographical dissimilarities, a standardized and appropriate volume of body fluids, methods of DNA extraction and diagnostic performance of HPV detection protocols [32].

Finding from this study showed that HPV-31, 35 and 16 were the most commonly identified genotypes among women in Nigeria. HPV-16 is considered as the most prominent type involved in the development of cervical cancer and other HPV-associated malignancies. HPV-35 is also an oncogenic HPV type, which is closely related phylogenetically to HPV-16. Some previous studies have shown that HPV-35 is the sixth most frequently detected HPV high-risk type in CIN3 and invasive cervical cancer [33,34]. Further analysis of women diagnosed with ASCUS through ICC by geopolitical zone in Nigeria confirmed that despite the differences in the prevalence of HPV-31, 35 and 16, these three genotypes remained the most common.

The prevalence of HPV-31 (70.8%), 35 (69.9%) and 16 (52.9%) was high. This could be because most of the women screened for cervical cancers are adults over 18 years of age, the high prevalence of these HPV types in the study population suggests the need for a robust measure to prevent future re-occurrence. A previous review on the burden of HPV infections in the extended Middle East and Northern Africa reported a low prevalence of HPV infection in women with normal cervical cytology, which is not in agreement with previous findings [35]. Also, it may be suggestive that several factors including sociodemographic and behavioural features of females could have influenced the broad range of views reported by dissimilar studies. The outcome of these studies could also be associated with the extent of adopting and executing preventive measures which include pap screening, promotion of contraceptives, and HPV immunization, which are instrumental in depleting the burden of HPV infection [36].

In regards to our findings, we observed variations in the prevalence of HPV infection among women across the studies which could be explained by the differences in the type of laboratory protocols used for HPV detection. This is because the detection rates were similar for HPV using PCR, hybridization, and PCR-hybridization, which were applied in 18 studies. Sample type may be one of the factors leading to differences in prevalence rates. Our analysis demonstrated that the detection rate of HPV using cytobrush/cervical brush for vaginal samples was significantly higher than the use of endocervical swab samples. Based on this, it could be inferred that among the various biological specimen, the vaginal sample provides the best performance for antigen, antibody, NAATs of HPV. This conforms with the report of Farahmand et al. [3].

HPV vaccines have proven to be harmless and efficacious by offering long-term protection against HPV infections [37]. Data from this systematic review suggests that currently available HPV vaccines could prevent a significant number of women in Nigeria. Unfortunately, Nigeria is currently not prepared to implement the National HPV vaccination despite the availability and use of these vaccines in some lower- and middle-income countries. This will consequently leave the majority of Nigerian women unprotected against HPV infection.

The main barriers militating against the use of HPV vaccinations in Nigeria include the long duration it would take for the vaccine to be ready for mass distribution, cost of procurement and inadequate infrastructure needed to deliver the vaccine to women at risk of contracting HPV infection and cervical cancer.

One of the major concerns in the use of HPV vaccines is that most polyvalent ones do not protect against HPV types other than 6, 11, 16, and 18. For instance, the majority of Female Sex Workers in the Netherlands were infected with HPV genotypes other than those was covered by the current vaccines [38]. To overcome this concern, it is recommended that the use of a nine-valent vaccine (9vHPV) instead of bivalent and quadrivalent vaccines be considered. The 9vHPV vaccine contains type-6, -11, -16, -18, -31, -33, -45, -52 and -58 which was approved by the USA Food and Drug Administration (FDA) in December 2014 and by the European Medicines Agency (EMA) in June 2015 [39].

Moreover, as a measure for ensuring early cervical cancer diagnosis, the use of screening tools such as cost-effective cervical screening through visual inspection with acetic acid and/or Lugol’s iodine, or detection of high-risk HPV types [40] could reduce the high burden of cervical cancer morbidity and mortality in Nigeria. The current WHO recommendations on cervical cancer screening are based on age of women. However, data from studies used for this systematic review revealed that the prevalence of HPV infection was similar within the three-age group and was not significantly associated with age. A dissimilar study accounted the effect of age on HPV prevalence in Africa [41]. The outcome of some other reports revealed the HPV infection prevalence increases inversely with age in younger females unlike the older counterparts where there is either an increase, a plateau or a decrease. This is not similar with a study that observed no influence of age on HPV prevalence [42].

Previous studies conducted in rural and semi-rural southwestern Nigeria revealed that HPV prevalence increased with decreased age in younger females [18]. In the other study, it was observed to directly vary with age in their older counterparts [43]. Although the broad range of outcomes of the effect of age on the prevalence of HPV is well-reported and suggests to a large extent the influence of dissimilarities in sexual behaviour of women of different demographic areas [28]. The difference between our result and previous studies on African or non-African women reveals distinct socioeconomic features and HPV risk factors of the populations surveyed.

## Conclusion

HPV prevalence is high in Nigeria and associated with several risk factors. Vaccination of women not infected with HPV 16/31/35 and rapid screening for high-risk HPV genotypes for the prevention of cervical cancer is recommended. The exceptionally high prevalence of HPV in the North-eastern part of Nigeria (an insurgency affect zone) requires urgent public health attention such as encouraging the use of cost-effective and acceptable tools to increase cervical cancer screenings and HPV DNA testing among the population to have an elaborate understanding of the prevalence of HPV genotypes and associated drivers. Moreover, the enrolment standard in some studies was biased as only women with possible illnesses (such as HIV/AIDS) underwent investigation which could have increased the HPV positivity rate. The limited sample size or absence of studies in some regions will not permit categorical inference on the association of HPV pooled prevalence and geographical regions of Nigeria.

## Data Availability

Being a systematic review article, the data synthesised and presented in the results section have been well referenced. However, raw data (in excel sheet) used in the statistical analysis will be made available on request through the corresponding author (I. N. Abdullahi).
